# Tumour cell thrombospondin-1 regulates tumour cell adhesion and invasion through the urokinase plasminogen activator receptor

**DOI:** 10.1054/bjoc.2000.1268

**Published:** 2000-07-03

**Authors:** D Albo, V L Rothman, D D Roberts, G P Tuszynski

**Affiliations:** Departments of Surgery and Pathology, MCP Hahnemann University, Philadelphia, PA, 19102, USA; Laboratory of Pathology, National Cancer Institute, Bethesda, MA, 20892, USA

**Keywords:** urokinase plasminogen activator, plasmin, pericellular proteolysis

## Abstract

We have previously shown that platelet-produced thrombospondin-1 up-regulates the urokinase plasminogen activator and its receptor and promotes tumour cell invasion. Although tumour cells produce thrombospondin-1 in vivo, they produce only minimal amounts of thrombospondin-1 in vitro. To determine the effect of tumour cell-produced thrombospondin-1 in the regulation of the plasminogen/plasmin system and tumour cell invasion, we studied *THBS-1* -transfected MDA-MB-435 breast cancer cells that overexpress thrombospondin-1. The role of urokinase plasminogen receptor in thrombospondin-1-mediated adhesion and invasion was studied by antisense inhibition, enzymatic cleavage and antibody neutralization. Tumour cell adhesion to collagen and laminin was evaluated. Tumour cell invasion was studied in a modified Boyden chamber collagen invasion assay. Tumour cell thrombospondin-1 induced a 2–7 fold increase in urokinase plasminogen activator receptor and cell-associated urokinase plasminogen activator expression and a 50–65% increase in cell-associated urokinase plasminogen activator and plasmin activities. Furthermore, tumour cell thrombospondin-1 promoted tumour cell invasion and decreased tumour cell adhesion through up-regulation of urokinase plasminogen activator receptor-controlled urokinase plasminogen activator and plasmin activities. We conclude that tumour cell-produced thrombospondin-1 may play a critical role in the regulation of tumour cell adhesion and tumour cell invasion. © 2000 Cancer Research Campaign


*British Journal of Cancer* (2000) **83**(3), 298–306
© 2000 Cancer Research Campaign
doi: 10.1054/ bjoc.2000.1268, available online at http://www.idealibrary.com on IDEAL®

### Tumour cell thrombospondin-1 regulates tumour cell adhesion and invasion through the urokinase plasminogen activator receptor

**D Albo[1], VL Rothman[1], DD Roberts[2] and GP Tuszynski[1]**

[1]Departments of Surgery and Pathology, MCP Hahnemann University, Philadelphia, PA 19102, USA; [2]Laboratory of Pathology, National Cancer Institute, Bethesda, MA 20892, USA

**Summary** We have previously shown that platelet-produced thrombospondin-1 up-regulates the urokinase plasminogen activator and its receptor and promotes tumour cell invasion. Although tumour cells produce thrombospondin-1 in vivo, they produce only minimal amounts of thrombospondin-1 in vitro. To determine the effect of tumour cell-produced thrombospondin-1 in the regulation of the plasminogen/plasmin system and tumour cell invasion, we studied *THBS-1*-transfected MDA-MB-435 breast cancer cells that overexpress thrombospondin-1. The role of urokinase plasminogen receptor in thrombospondin-1-mediated adhesion and invasion was studied by antisense inhibition, enzymatic cleavage and antibody neutralization. Tumour cell adhesion to collagen and laminin was evaluated. Tumour cell invasion was studied in a modified Boyden chamber collagen invasion assay. Tumour cell thrombospondin-1 induced a 2–7 fold increase in urokinase plasminogen activator receptor and cell-associated urokinase plasminogen activator expression and a 50–65% increase in cell-associated urokinase plasminogen activator and plasmin activities. Furthermore, tumour cell thrombospondin-1 promoted tumour cell invasion and decreased tumour cell adhesion through up-regulation of urokinase plasminogen activator receptor-controlled urokinase plasminogen activator and plasmin activities. We conclude that tumour cell-produced thrombospondin-1 may play a critical role in the regulation of tumour cell adhesion and tumour cell invasion. © 2000 Cancer Research Campaign

**Keywords**: urokinase plasminogen activator; plasmin; pericellular proteolysis

Tumour cell invasion is a complex, multistep process that involves the controlled degradation of the extracellular matrix by tumour cell-associated proteases (Mignatti, 1993). Generation of plasmin on the tumour cell surface is believed to play a crucial role in the process of invasion. Tumour cell-bound plasmin directly degrades the tumour-associated stroma, activates precursors of proteases (i.e. matrix metalloproteinases), and activates latent growth factors stored in the extracellular matrix (i.e., transforming growth factor-β1). These growth factors then act in a paracrine fashion, further promoting tumour cell growth and invasion (Testa and Quigley, 1990; Vassalli, 1991). While pericellular proteolysis is crucial for tumour cell invasion to occur, excessive proteolysis inhibits invasion, presumably by destroying the matrix scaffold that tumour cells need in order to adhere and migrate (Krishnamurti, 1992; Hosokawa et al,, 1993). Thus, a controlled proteolysis, with an adequate temporal and spatial expression of proteases, has to take place for the process of tumour cell invasion to occur.

One of the main components of the plasminogen/plasmin system is the urokinase plasminogen activator receptor (uPAR) (Vassalli, 1985; Plow, 1986; Blasi, 1990; Roldan, 1990). uPAR, a member of a family of cysteine-rich cell surface proteins, lacks a transmembrane domain. It is anchored to the cell surface by a glycosylphosphatidylinositol (GPI) linkage that is synthesized concurrently to the postranslational removal of the carboxy-terminal signal sequence (Ploug et al, 1993). Experimental data has demonstrated that uPAR plays a critical role in tumour invasion and metastasis (Blasi, 1993*a*; 1993*b*). In a series of elegant experiments, inhibition of uPAR resulted in prevention of metastasis, tumour growth and angiogenesis in different in vitro and in vivo models (Ossowski et al, 1991; Ossowski, 1992; Kariko et al, 1993; Mohanam et al, 1993; Kook et al, 1994; Mohanam et al, 1994; Yu et al, 1997). Furthermore, it has been shown that high levels of uPAR in primary tumours correlate with their malignant potential, therefore serving as a prognostic indicator (Graeff, 1992; Costantini et al, 1996). uPAR binds urokinase plasminogen activator (uPA) in a saturable, reversible, and specific manner, with a Kd of 0.1–1 nM and maximal binding of $1 \times 10^4$–$2.2 \times 10^5$ sites per cell (high affinity and low capacity) (Barnathan, 1990). uPA is the main cell-associated activator of plasmin and it has been shown to play a key role in tumour invasion and metastasis (Testa and Quigley, 1990; Ellis, 1991; Ossowski et al, 1991; Blasi, 1993*a*; 1993*b*; Mignatti, 1993). uPA levels in primary tumours have been shown to function as a prognostic indicator (Foekens, 1992).

While degradation of the extracellular matrix is necessary for tumour cell invasion, excessive matrix degradation has been shown to inhibit tumour cell migration and prevent invasion (Krishnamurti, 1992; Hosokawa et al, 1993; Albo et al, 1994; 1997). PAI-1, the main inhibitor of uPA, has been shown to localize to the extracellular matrix in several malignancies in

*Received 16 September 1999*
*Revised 8 March 2000*
*Accepted 10 April 2000*

*Correspondence to:* GP Tuszynski association with matrix proteins such as vitronectin (Seiffert, 1994; Bianchi, 1995). PAI-1 has been shown to promote breast tumour cell invasion by preventing excessive matrix degradation by tumour-associated plasmin-mediated proteolytic activity (Albo et al, 1997). Considerable evidence indicates that increased levels of tumour-associated PAI-1 are a strong, independent prognostic indicator of poor clinical outcome in cancer patients (Costantini et al, 1996; Grondahl-Hansen et al, 1997).

Several extracellular matrix proteins have been shown to modulate tumour growth and invasion in a paracrine fashion (Laiho, 1989; Keski-Oja, 1991; Tuszynski et al, 1997). TSP-1 (encoded by the *THBS-1* gene), a large glycoprotein that localizes to the extracellular matrix in several different tumours, has been shown to play a critical role in tumour progression and invasion (Tuszynski, 1993). TSP-1 has been shown to promote tumour cell adhesion, migration, invasion and angiogenesis, crucial steps in the process of metastasis (Tuszynski et al, 1987*a*; 1987*b*; Clezardin et al, 1991; Yabkowitz et al, 1993). Our laboratory has shown that platelet-produced TSP-1 up-regulates uPA and uPAR, increases plasmin-mediated proteolytic activity in different tumour cells and promotes tumour cell invasion. Furthermore, we have recently shown that neutralization of uPA binding to uPAR at the tumour cell surface with antibodies against either uPA or uPAR results in inhibition of platelet TSP-1-mediated tumour cell invasion in vitro (Albo et al, 1994; 1997; Arnoletti, 1995; Wang et al, 1996*a*; 1996*b*).

Our previous findings supporting a role for TSP-1 in tumour cell invasion were generated using human platelet-produced TSP-1 (Wang et al, 1995; 1996*c*; Albo et al, 1997; Albo, 1998). A potential pitfall of these findings is that TSP-1 is a very large glycoprotein, and, therefore, although platelet produced-TSP-1 is likely to play a role in the haematogenous phase of tumour spread, it is not likely to achieve significant concentrations at the tissue level. Although no significant changes in the structure of platelet-produced and tumour-produced TSP-1 have been reported, tumour-produced TSP-1 could have different properties than platelet-produced TSP-1 in terms of the regulation of the plasminogen/plasmin system and tumour cell invasion. In addition, tumour cells produce TSP-1 in vivo through a paracrine interaction with activated stromal cells, and, therefore, produce small amounts of TSP-1 when cultured in vitro. In this study we used breast tumour cells transfected with full-length *THBS-1* cDNA that overexpress TSP-1.

Additionally, our previously published data supporting a role for the plasminogen/plasmin system in TSP-1-mediated tumour cell invasion was primarily based on antibody neutralization studies (Albo et al, 1997; Albo, 1998). The potential problems associated with the use of antibodies are related to their specificity and the fact that most of the uPA is bound to uPAR on the cell surface and, therefore, either uPA and/or uPAR may be protected from inactivation by their respective antibodies. Additionally, the antibodies may induce clustering of the receptors and therefore induce spurious activation of the receptors and their signalling pathways. Therefore, we developed three other strategies to study the role of the plasminogen/plasmin system in TSP-1-mediated tumour cell invasion in addition to the use of neutralizing antibodies: a) removal of uPAR from the cell surface by cleaving its GPI anchor with GPI-specific phospholipase C; b) inhibition of uPAR production at the mRNA level by using uPAR antisense technology; c) inhibition of plasminogen binding with ε-aminocaproic acid, a plasminogen receptor antagonist.

### MATERIALS AND METHODS

### Materials

All reagents, unless otherwise specified, were purchased from Sigma Chemical Company (St. Louis, MO, USA). The human breast adenocarcinoma cell line MDA-MB-435 was purchased from the American Type Culture Collection (HTB 26, Rockville, MD, USA). MDA-MB-435 cell line was established from pleural effusions of a patient with metastatic breast cancer. These cells form invasive tumours in nude mice consistent with a breast primary. Tissue culture supplies were purchased from Fisher Scientific Company (Pittsburgh, PA, USA).

### THBS-1 transfection

Transfection of MDA-MB-435 cells with the *THBS-1* expression plasmid or unaltered pCMVBamNeo vector using a calcium phosphate-based kit (GIBCO-BRL, Gaithersburg, MD, USA) was previously described (Weinstat-Saslow et al, 1994).

### Cell clones

Three clones were selected for this study: TH5 cells (vector alone, control); TH26 cells (7.5 × higher TSP-1 expression than TH5 cells); TH29 cells (4 × higher TSP-1 expression than TH5 cells). All three clones were previously characterized (Weinstat-Saslow et al, 1994). TSP-1 production by the different clones used was assessed by Western blot and TSP-1 ELISA.

### Cell culture

TH5, TH26 and TH29 cells were cultured in Dulbecco's Modified Eagle's Medium (DMEM) supplemented with 10% fetal calf serum, 100 units $ml^{-1}$ of penicillin, 100 μg $ml^{-1}$ of streptomycin, and 750 μg $ml^{-1}$ G418 (GIBCO-BRL). The cultures were maintained on plastic and incubated in 5% $CO_2$–95% air at 37°C in a humidified incubator. Cell cultures were noted to be pathogen-free. Tumour cells were harvested from subconfluent cultures (80–90% confluence) by short exposure to 0.02% ethylenediamine tetraacetic acid (EDTA) solution and re-suspended in serum-free DMEM before use.

### Antisense uPAR transfection

The uPAR antisense construct (LKAS) was previously described (Yu et al, 1997). Briefly, it consists of a 296-bp uPAR antisense cDNA fragment subcloned into a BamHI- and HindIII-digested LK444 vector under a β-actin promoter. Sequence analysis of the fragment showed 100% homology with the published uPAR sequence. After three in vitro passages, TH26 cells (7.5 × higher TSP-1 expression than control TH5 cells) were plated at $3 \times 10^5$ cells per well of a six-well plate, and when 80% confluent, they were transiently transfected for 6–8 h with 1.5 μg of LK444 (designated LK444–TH26) or LKAS (designated LKAS-TH26) using 0, 6, 9, 12, or 20 μl of Lipofectin (Gibco Laboratories, Rockville, MD, USA) in serum-free DMEM according to manufacturer's protocol. After transfection, the cells were incubated for 24 h in DMEM supplemented with 10% FCS. The cells were then incubated for 24 h in serum-free media. At the end of the incubation period, tumour cell media were obtained. The cells were

© 2000 Cancer Research Campaign washed with phosphate buffered saline (PBS) and tumour cell extracts were harvested by adding cold 1% Triton X-100 in TBS, pH 8.5. The extracts were centrifuged at 10 000 rpm to remove cell debris. Quantitation of uPAR and uPA and measurement of uPA and plasmin activity were performed as described below.

### Quantitation of uPAR, uPA, PAI-1, TGF-β1 and TSP-1

TH5, TH26, or TH29 cells were plated in six-well plates and allowed to attach and grow to 80–90% confluency in FCS-containing medium. The cells were weaned from the FCS-containing media to a serum-free media over 24 h. The cells were then incubated for 48 h at 37°C in DMEM supplemented with 0.1% bovine serum albumin (BSA), 100 units $ml^{-1}$ of penicillin, 100 μg $ml^{-1}$ of streptomycin, and 750 μg $ml^{-1}$ G418 with and without the addition of 100 μg $ml^{-1}$ of either polyclonal goat anti-human TSP-1 neutralizing antibody or control goat IgG. At the end of the incubation period, tumour cell media and extracts were obtained as described above. uPAR levels in cell extracts were measured using the IMUBIND total uPAR enzyme-linked immunosorbent assay kit (American Diagnostica Inc, Greenwich, CT, USA). uPA concentration in the cell media and extracts from the different treatment groups was measured using the IMUBIND total uPA enzyme-linked immunosorbent assay kit (American Diagnostica Inc). PAI-1 concentration in the cell media from the different treatment groups was measured using the IMUBIND total PAI-1 enzyme-linked immunosorbent assay kit (American Diagnostica Inc). TSP-1 concentrations were measured by a competitive TSP-1 immunosorbent assay developed in our laboratory and previously described (Tuszynski et al, 1987*c*). The lower detection limits of the assays are 0.1–2.0 ng total uPAR/uPA/PAI-1/TSP-1 per ml of sample. Samples of equal amounts of total protein (as determined by BCA protein analysis) were evaluated. Assay procedures were performed according to the vendor's instructions. Total uPAR, uPA, PAI-1, and TSP-1 levels were quantitated by measuring solution absorbencies and comparing the values with those of a standard curve. Results shown are the average of three separate determinations. Total and activated TGF-β1 levels in the conditioned media were measured by the Quantikine human TGF-β1 ELISA assay (R&D Systems, Minneapolis, MN, USA) following the manufacturer's protocol.

### Western immunoblotting

Tumour cell extract protein concentration was determined by BCA protein analysis (Bio-Rad Laboratories, Richmond, CA, USA). Samples of equal amount (approximately 30 μg of total protein per lane) were fractionated on 8–25% gradient SDS-PAGE and then transferred to nitrocellulose membranes (Micron Separations Inc, Westborough, MA, USA) using the Pharmacia phast-gel electrophoresis system. Nonspecific binding was blocked with 5% milk in PBS containing 0.05% Tween 20 (PBS-T) overnight. The immunoblots were then incubated with either 5 μg $ml^{-1}$ of polyclonal rabbit anti-human uPAR or 80 μg $ml^{-1}$ polyclonal goat anti-human TSP-1 IgG for 1 h at room temperature in PBS-T. After washing, the immunoblots were incubated with horseradish peroxidase-conjugated anti-rabbit or anti-goat IgG for 45 min. The bound antibodies were detected using an enhanced chemiluminescence (ECL) system (Amersham, Arlington Heights, IL, USA).

### uPA and plasmin activities uPA and plasmin enzymatic activities were determined spectrophotometrically using uPA or plasmin-specific chromogenic substrate assays (Spectrozyme UK, American Diagnostica Inc). Enzymatic levels were measured directly in microtitre plates at 405 nm.

### Tumour cell invasion assay

Tumour cell invasion was investigated in a modified Boyden chamber assay. This assay was employed since it has been demonstrated that invasiveness in this assay correlates well with the metastatic potential of a given cell line in vivo (Albini, 1987; Wang et al, 1996*a*; 1996*b*). Polycarbonate filters, 8 μg pore size (Millicell, Millipore Corporation, Bedford, MA, USA), were coated with 100 μg type I collagen (1 mg $ml^{-1}$ in 60% ethanol) and dried overnight at 25°C. Blind-well Boyden chambers were filled with 600 μl of DMEM containing 0.1% BSA in the lower compartment, and the coated filters were mounted in the chamber. Approximately 50 000 TH5, TH26, TH29, LK444-TH26, or LKAS-TH26 tumour cells (tested to be greater than 95% viable) suspended in 400 μl serum-free medium were placed in the upper chamber of the apparatus and allowed to settle onto the collagen-coated membrane. After an incubation period of 6 h at 37°C (previously established in our laboratory), the cells on the upper surface of the filter were removed with a cotton swab. The filters were then fixed in 3% glutaraldehyde solution and stained with 0.5% crystal violet solution. Invasive cells adhering to the undersurface of the filter were counted using a phase contrast microscope (× 400). The data were expressed as the summation of the number of invasive tumour cells in five representative fields. All experiments were performed in triplicate.

### In vitro tumour cell invasion inhibition assays

TH26 tumour cell invasion was also determined with the addition to the upper chamber of either: a) anti-uPA antibody (10 μg $ml^{-1}$); b) anti-uPAR antibody (10 μg $ml^{-1}$); c) GPI-specific phospholipase C (PLC, 1 IU $ml^{-1}$), an enzyme that cleaves the GPI anchor that attaches uPAR to the tumour cell surfaces or; d) ε-aminocaproic acid (ACA, 0.25 M), a lysine analogue that prevents formation of plasmin at the cell surface by blocking plasminogen binding (Hajjar, 1995). The number of invasive cells was counted as described above for the cell invasion assay. Cleavage of uPAR from the tumour cells by PLC was confirmed by measuring uPAR on tumour cell extracts and media by ELISA as previously described.

### Tumour cell adhesion assay

Tumour cell adhesion to different substrates was studied as previously described (Tuszynski and Murphy, 1990). Briefly, 50 μl of either a 40 μg $ml^{-1}$ solution of collagen type I, collagen type IV, laminin, or bovine serum albumin (BSA, negative control) dissolved in DMEM, pH 7.3, were placed in 96-well microtitre wells and dried overnight at 25°C. The wells were washed with PBS and then treated with 200 μl PBS containing BSA for 1 h to block non-specific binding and then washed three more times with PBS. Cells were harvested, washed and suspended in serum-free DMEM. 20 000 TH5, TH26, or LKAS-TH26 tumour cells were

© 2000 Cancer Research Campaign added to each well and incubated in serum-free DMEM for 1 h at 37°C in a $CO_2$ incubator. Additionally, 20 000 TH26 tumour cells treated with 0.25 M ε-aminocaproic acid were also plated and incubated for 1 h. Non-adherent cells were removed by aspiration and the wells washed three times with PBS. The total cell-associated protein was determined by dissolving the attached cells directly in the microtiter wells with 200 µl of the Pierce BCA working solution (Pierce Chemical Co, Rockford, IL, USA) and the absorbance of each well was determined at 562 nm with a microtitre reader plate.

### Statistical analysis

All experiments were done in triplicate and performed at least twice. Values are expressed as the mean ± standard deviation. Statistical analysis was performed using Sigmastat software (Jandel Corporation, San Rafael, CA, USA). Where several groups were compared to a control group, analysis of variance (ANOVA) and Student–Newman–Keuls method were utilized. $P$ values less than 0.05 were considered significant. Dose-dependent response was evaluated by linear regression analysis.

### RESULTS

### TSP-1 expression

TH5, TH29, and TH26 cells have been previously characterized (Weinstat-Saslow et al, 1994). TSP-1 expression by TH5, TH29, and TH26 cells used in this study was confirmed by Western immunoblot analysis (Figure 1). When media conditioned by these cells for 48 h were analysed for TSP-1 by ELISA, we found that TH26 (4.9 µg ml$^{-1}$) and TH29 (2.6 µg ml$^{-1}$) cells showed a 7.5- and 4-fold increase respectively, in TSP-1 expression compared to control TH5 cells (0.66 µg ml$^{-1}$).

### TGF-β1 expression

Variable levels of total (active + inactive) TGF-β1 were observed in our different cell lines. Total TGF-β1 expression was 210 ± 12 pg 10$^{-1}$ tumour cells by TH26 cells, 54 ± 3 pg 10$^{-6}$ tumour cells by TH29 cells and 84 ± 5 pg 10$^{-6}$ tumour cells by TH5 cells. However, active TGF-β1 levels were higher in the low-TSP-1 producing TH5 cells (75 ± 8 pg 10$^{-6}$ tumour cells) than in intermediate TSP-1 producing TH29 cells (48 ± 2 pg 10$^{-6}$ tumour cells) or in the high TSP-1-producing TH26 cells (58 ± 5 pg 10$^{-6}$ tumour cells).

### The effect of tumour cell-produced TSP-1 on uPAR expression

TH26 cells and TH29 cells expressed higher uPAR concentrations in a manner that was directly proportional to the amount of TSP-1 produced by these cells, as demonstrated by ELISA and Western immunoblotting (Figures 2 and 3). TH5 cells (control), expressed 6.74 ± 0.12 ng ml$^{-1}$ of uPAR. TH26 cells expressed 34.80 ± 0.12 ng ml$^{-1}$ of uPAR (5-fold increase). TH29 cells expressed 12.75 ± 0.19 ng ml$^{-1}$ of uPAR (2-fold increase). The differences in uPAR expression between the TH26 or TH29 cells and the TH5 cells were statistically significant ($P$ < 0.01). Addition of neutralizing anti-TSP-1 antibody to the high TSP-1-producing TH26 cells significantly decreased uPAR expression by these cells (9.99 ± 0.37 ng ml$^{-1}$, $P$ < 0.01 vs TH26 cells alone). Control IgG showed no effect on TH26 cell uPAR expression (40.3 ± 1.83 ng ml$^{-1}$).

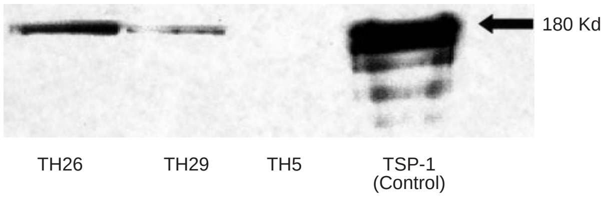

**Figure 1** Thrombospondin-1 expression in transfected cell lines. TH5 (transfected with the vector alone), TH26 (*THBS-1*-transfected; high TSP-1 producer), or TH29 (*THBS-1*-transfected; low TSP-1 producer) breast cancer cells were grown to 80–90% confluency in serum-containing media and then weaned to serum-free media over a 24 h period. After a 24-h incubation in serum-free media, tumour cell media from the different cell types were obtained. Samples of equal amount were fractionated on 8–25% gradient SDS-PAGE and then transferred to a nitrocellulose membrane. Nonspecific binding was blocked with 5% milk in PBS-T overnight. The immunoblot was then incubated with 80 µg ml$^{-1}$ polyclonal goat anti-human TSP-1 IgG for 1 h at room temperature in PBS-T. After washing, the immunoblots were incubated with horseradish peroxidase-conjugated anti-goat IgG for 45 min. The bound antibodies were detected using an enhanced chemiluminescence system. The 180 Kd band represents non-cleaved TSP-1.

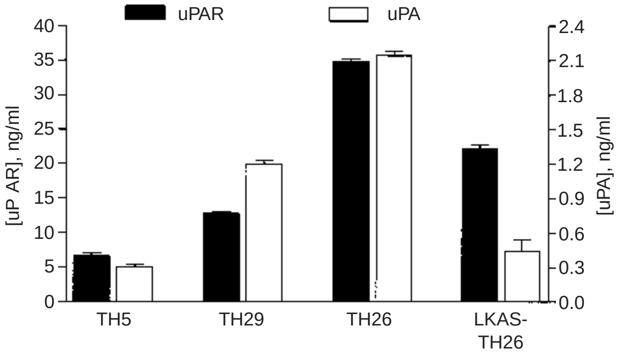

**Figure 2** uPAR and uPA expression in transfected cell lines. TH5, TH26, TH29, or LKAS-TH26 cells were grown to 80–90% confluency on FCS-containing medium. The cells were weaned off the FCS-containing media to a serum-free media over 24 h. After a 48-h incubation period, tumour cell extracts were obtained by addition of cold 1% Triton X-100. uPAR and uPA expression were measured by ELISA.

### The effect of tumour cell-produced TSP-1 on uPA expression

While total uPA production (tumour cell extract + tumour cell media) was not significantly changed in the TH5, TH26, and TH29 cells (data not shown), uPA expression on the tumour cell extracts was 7-fold higher in the TH26 cells and 4-fold higher in the TH29 cells compared to the control TH5 cells (Figure 2). TH5 cells (control), expressed 0.30 ± 0.12 ng ml$^{-1}$ of uPA. TH26 cells expressed 2.14 ± 0.18 ng ml$^{-1}$ of uPA. TH29 cells expressed 1.20 ± 0.33 ng ml$^{-1}$ of uPA. The differences in uPA expression between the TH26 or TH29 cells and the TH5 cells were statistically significant ($P$ < 0.04). Addition of neutralizing anti-TSP-1 antibody to the high TSP-1-producing TH26 cells significantly decreased uPA expression by these cells (0.40 ± 0.10 ng ml$^{-1}$, $P$ < 0.01 vs TH26 cells alone). Control IgG showed no effect on TH26 cell uPAR expression (1.98 ± 0.08 ng ml$^{-1}$).

© 2000 Cancer Research Campaign

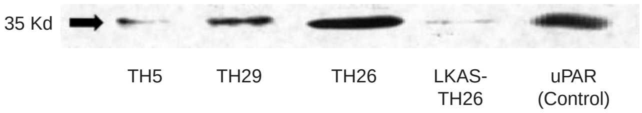

**Figure 3** uPAR expression in transfected cell lines. Tumour cell extracts were obtained as outlined in Figure 2. Additionally, TH26 cells were transiently co-transfected with the LKAS uPAR antisense construct (cells named LKAS-TH26) for 6–8 h. After a 36-h incubation in serum-containing media, tumour cells were placed in serum-free media for 24 h. At that time, tumour cell extracts were obtained by addition of cold 1% Triton X-100. uPAR expression in tumour cell extracts was determined by Western immunoblot analysis by using a polyclonal rabbit anti-human uPAR primary antibody and the corresponding peroxidase-conjugated anti-rabbit IgG secondary antibody. The bound antibodies were detected using an enhanced chemiluminescence system.

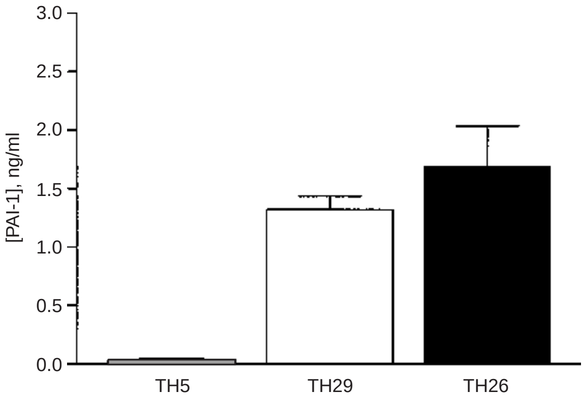

**Figure 4** PAI-1 levels in transfected cells. TH5, TH26, or TH29 cells were grown to 80–90% confluency on FCS-containing medium. The cells were weaned off the FCS-containing media to a serum-free media over 24 h. After a 48-h incubation period, tumour cell conditioned media was harvested. PAI-1 levels were measured by ELISA.

### The effect of tumour cell-produced TSP-1 on PAI-1 expression

PAI-1 expression was significantly up-regulated by tumour cell TSP-1 (Figure 4). While TH5 cells expressed only 0.04 ± 0.02 ng $ml^{-1}$ of PAI-1 in their cell media, TH29 cells expressed 1.32 ± 0.20 ng $ml^{-1}$ and TH26 cells expressed 1.69 ± 0.60 ng $ml^{-1}$. The difference in PAI-1 expression between TH5 cells and TH29 or TH26 cells was statistically significant ($P < 0.03$). The difference in PAI-1 expression between TH26 and TH29 cells was not statistically significant ($P = 0.25$).

### The effect of tumour cell-produced TSP-1 on uPA and plasmin activities

Tumour cell-associated uPA and plasmin activities were higher in the TSP-1-producing tumour cells in a manner that was directly proportional to the amount of TSP-1 produced (Figure 5). uPA and plasmin activity were 50–65% higher in the high TSP-1-producing tumour cells (TH26 cells) than in the control cells (TH5 cells). The differences in uPA or plasmin activity between the TH26 and the TH5 cells were statistically significant ($P < 0.01$).

### The effect of LKAS transfection on TH26 uPAR expression

Transient uPAR antisense transfection reduced uPAR expression on TH26 cells by 50–70% (Figures 2 and 3). Reduction of uPAR

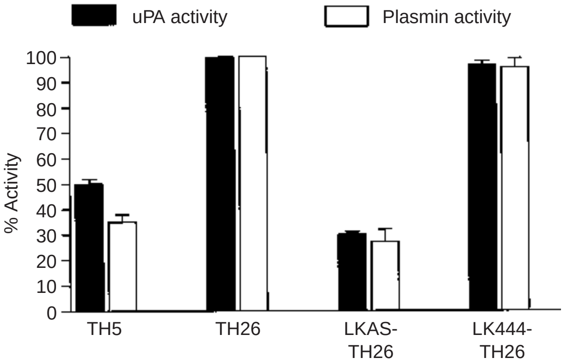

**Figure 5** uPA and plasmin activities in transfected cell lines. Tumour cell extracts were obtained as outlined in Figure 3. uPA and plasmin activities were measured by using uPA or plasmin-specific chromogenic substrate assays. Antisense uPAR transfection (LKAS-TH26 cells) resulted in a 70% inhibition of plasmin and uPA activities on TH26 cells. Vector control transfection (LK444–TH26) showed no reduction in activity compared to TH26 controls.

expression in the tumour cell extracts was proportional to the dose of lipofectin used as demonstrated by linear regression analysis ($R = 0.874$). Maximum inhibition of uPAR expression was achieved with 6 µl of lipofectin per 1.5 µg of DNA. Concentrations greater than 12 µl of lipofectin per 1.5 µg of DNA compromised cell viability without significantly reducing uPAR expression on the tumour cells (data not shown). LK444-TH26 (vector control transfected cells) cell extracts expressed similar amounts of uPAR than the non-transfected tumour cells (33.90 ± 2.69 vs 35.50 ± 1.70 ng $ml^{-1}$ of uPAR, respectively, $P = 0.67$).

### The effect of LKAS transfection on TH26 uPA expression

Although no change in total uPA production (cell extract + media) was observed, uPA expression was reduced by 50–70% in the LKAS-TH26 cell extracts (Figure 2). Reduction of uPA expression in the tumour cell extracts was proportional to the dose of lipofectin used as demonstrated by linear regression analysis ($R = 0.870$). Maximum inhibition of uPA expression was achieved with 6 µl of lipofectin per 1.5 µg of DNA. Concentrations greater than 12 µl of lipofection per 1.5 µg of DNA compromised cell viability without significantly reducing uPA expression on the tumour cells (data not shown). LK444-TH26 (vector control transfected cells) cell extracts expressed similar amounts of uPA than the non-transfected tumour cells (2.18 ± 0.10 vs 2.32 ± 0.05 ng $ml^{-1}$ of uPA, respectively, $P = 0.33$).

### The effect of LKAS transfection on TH26 uPA and plasmin activities

Antisense uPAR transfection reduced uPA and plasmin activities on TH26 tumour cells (Figure 5). uPA activity was reduced by 70% on LKAS-TH26 tumour cells compared to control (LK444-TH26 cells). Plasmin activity was reduced by 73% on LKAS-TH26 tumour cells compared to control (LK444-TH26 cells). The differences in uPA or plasmin activity between the LKAS-TH26 and the LK444–TH5 cells were statistically significant ($P < 0.001$).

 © 2000 Cancer Research Campaign

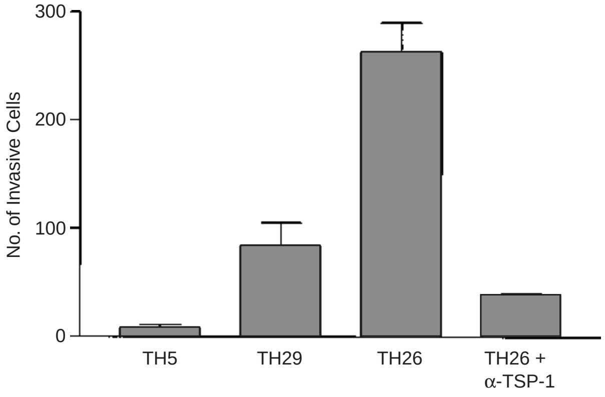

**Figure 6** Tumour cell invasion by transfected cell lines. 50 000 tumour cells were plated per well of a modified Boyden chamber collagen invasion assay. After a 6-h incubation period the number of cells traversing the membrane were counted and expressed as number of invasive cells per five high-power fields.

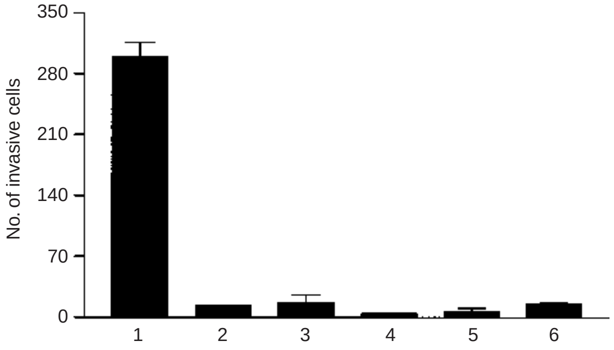

**Figure 7** The role of uPAR on TSP-1-mediated tumour cell invasion. 50 000 tumour cells were plated per well of a modified Boyden chamber collagen invasion assay. Additionally, TH26 cells were incubated in the presense of anti-TSP-antibody. After a 6-h incubation period the number of cells traversing the membrane were counted and expressed as number of invasive cells per five high-power fields. (1) TH26 tumour cells alone. (2) TH26 + neutralizing anti-uPAR antibody. (3) TH26 + neutralizing anti-uPA antibody; (4) ε-aminocaproic acid-treated TH26 cells. (5) TH26 + GPI-phospholipase C. (6) LKAS-TH26 (uPAR antisense co-transfected TH26 cells) tumour cells alone.

### The effect of tumour cell-produced TSP-1 in tumour cell invasion

Tumour cell invasion correlated with the amount of TSP-1 produced by the different cell lines (Figure 6). TH5 cells showed 8.0 ± 1.0 invasive cells per five high-power fields. TH26 cells showed 263.5 ± 46.5 invasive cells per five high-power fields (47.5-fold increase). TH29 cells showed 84.0 ± 21.4 invasive cells per five high-power fields (10.5-fold increase). The differences in tumour cell invasion between the TH26 or TH29 cells and the TH5 cells were statistically significant ($P < 0.05$). Addition of neutralizing antibody against TSP-1 significantly decreased the invasive capacity of TH26 cells (39 ± 2 invasive cells per five high-power fields, $P < 0.01$ vs TH26 cells alone). Control IgG did not significantly reduce the invasive capacity of TH26 cells (251 ± 27.5 invasive cells per five high-power fields, $P = 0.12$ vs TH26 cells alone).

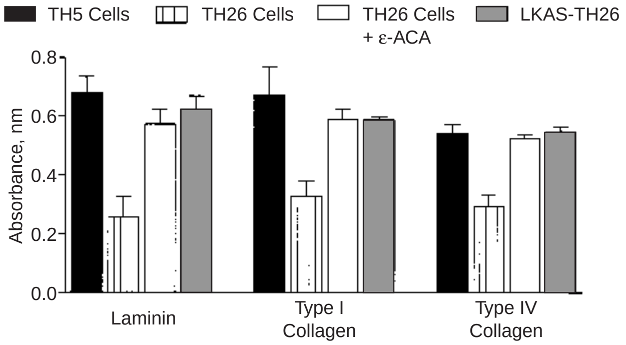

**Figure 8** The role of uPAR on tumour cell adhesion. Approximately 20 000 TH5, TH26 (with and without treatment with ε-aminocaproic acid) or LKAS-MDA tumour cells were plated per well of a 96-well microtitre plate pre-coated with either laminin, type I, or type IV collagen. After a 1-h incubation period the wells were washed with PBS. Total cell-associated protein was determined by dissolving the attached cells directly in the microtitre wells with 200 µl of the Pierce BCA working solution (Pierce Chemical Co) and the absorbance of each well was determined at 562 nm with a microtitre reader plate. LKAS-TH26: antisense uPAR transfected TH26 cells. ACA: ε-aminocaproic acid.

### The role of the plasminogen/plasmin system in tumour cell TSP-1-mediated tumour cell invasion

To determine the role of the plasminogen/plasmin system on tumour cell TSP-1-mediated tumour cell invasion, different antagonists were used (Figure 7). TH26 tumour cell invasion (380.0 ± 27.0 invasive cells per five high-power fields) was inhibited by blocking uPAR or uPA with neutralizing antibodies (13.5 ± 0.5 and 17.5 ± 8.5 invasive cells per five high-power fields, respectively), cleaving uPAR from the cell surface with GPI-specific phospholipase C (4.5 ± 0.5 invasive cells per five high-power fields), or blocking plasminogen binding to the cell surface with the lysine analogue ε-aminocaproic acid (7.5 ± 3.5 invasive cells per five high-power fields). Furthermore, a comparable inhibition on TH26 tumour cell invasion was achieved by transiently transfecting the TH26 cells with LKAS (uPAR antisense construct) (16.5 ± 0.5 invasive cells per five high-power fields). The differences in tumour cell invasion between TH26 cells alone and TH26 cells plus the addition of α-uPAR, α-uPA, GPI-PLC, ε-ACA, or LKAS were statistically significant ($P < 0.005$). Transfection of TH26 cells with LK444 (vector control) had no statistically significant effect on TH26 tumour cell invasion (286.0 ± 16.0 invasive cells per five high-power fields, $P = 0.10$).

ELISA confirmed that treatment with GPI-PLC stripped the TH26 tumour cells of uPAR. uPAR expression on non-treated TH26 tumour cell extracts and media was 35.99 ± 1.18 ng $ml^{-1}$ and 0.20 ± 0.50 ng $ml^{-1}$, respectively. uPAR expression on GPI-PLC-treated TH26 tumour cell extracts and media was 0.27 ± 0.07 ng $ml^{-1}$ and 3.5 ng $ml^{-1}$, respectively.

### The effect of tumour cell-produced TSP-1 in tumour cell adhesion

Tumour cell adhesion was inhibited by tumour cell-produced TSP-1 (Figure 8). A 2-fold decrease in TH26 tumour cell adhesion to laminin, type I collagen, and type IV collagen was observed compared to the TH5 tumour cells. The differences in tumour cell adhesion to the different substrates between the TH26 and the TH5 cells were statistically significant ($P < 0.01$). Antisense uPAR

*© 2000 Cancer Research Campaign*

transfection restored the adhesive capacity of the TH26 cells, showing that up-regulation of uPAR plays a central role in the decreased cell adhesion observed in the high TSP-1 producing cells (Figure 8). In order to determine whether this effect was related to some intrinsic anti-adhesive effects of the uPAR molecule or related to an enhanced generation of plasmin at the cell surface, TH26 cells were treated with ε-aminocaproic acid. Blocking binding of plasminogen to the TH26 tumour cell surface with ε-aminocaproic acid also restored the adhesive capacity of the TH26 tumour cells to control levels (Figure 8). These data show that the TSP-1-mediated decrease in tumour cell adhesion involves not only up-regulation of uPAR expression but also the conversion of plasminogen to plasmin at the tumour cell surface.

### DISCUSSION

Pericellular proteolysis plays a crucial role in tumour cell invasion. The controlled degradation of the extracellular matrix by tumour-associated proteases allows tumour cells to invade and metastasize (Mignatti, 1993). Plasmin is believed to play a central role not only as a proteolytic enzyme capable of cleaving most of the extracellular matrix components but also by activating inactive precursors of other key proteases (i.e. matrix metalloproteinases) produced by tumour cells and/or tumour-associated stromal cells. Additionally, during matrix degradation, plasmin can activate latent growth factors (i.e. TGF-β1) stored in the matrix that have potent effects on tumour growth and can provide for an amplification loop by up-regulating plasmin generation as well (Testa and Quigley, 1990; Vassalli, 1991).

We have previously established that platelet-produced TSP-1 promotes tumour cell invasion through up-regulation of the key regulatory components of the plasminogen/plasmin system, uPAR, uPA, and PAI-1 (Albo et al, 1997). Degradation of the extracellular matrix in the pericellular environment, such as the one promoted by uPAR-bound uPA, allows tumour cells to detach from the main colony and to invade surrounding tissues. By preventing excessive plasmin-mediated degradation of the extracellular matrix, PAI-1 has been shown to play a crucial role in tumour cell invasion and metastasis (Bianchi, 1995; Costantinin et al, 1996; Albo et al, 1997; Grondahl-Hansen et al, 1997). Furthermore, although a study in bladder cancer suggested that TSP-1 levels in the extracellular matrix inversely correlated to tumour growth (Grossfeld et al, 1997), several studies in breast, head and neck cancers have demonstrated a direct correlation between high TSP-1 and/or TSP-1 receptor expression and metastatic potential of the tumours (Wong et al, 1992; Clezardin et al, 1993; Arnoletti et al, 1994; Tuszynski and Nicosia, 1994; Roth et al, 1997). The TSP-1-mediated up-regulation of the plasminogen/plasmin system may in part be responsible for the higher metastatic potential in patients with higher TSP-1 levels. Additionally, higher blood levels of TSP-1 have been shown in patients with several malignancies, with patients with higher circulating levels of TSP-1 showing poorer outcomes (Tuszynski et al, 1992; Nathan et al, 1994; Yamashita et al, 1998).

Due to its large molecular weight, it is unlikely that the TSP-1 produced in the vascular compartment achieves significant tissue concentration. Although platelet-produced TSP-1 may indeed play a key role in the haematogenous spread of tumour cells, it is not likely to play a role in tissue invasion. Tumours produce TSP-1 in vivo through a tightly controlled paracrine interaction between tumour cells and stromal cells (i.e. tumour-associated fibroblasts). This paracrine interaction produces a very precise pattern of TSP-1 expression in vivo, with TSP-1 being stored in the matrix, and its CSVTCG-specific receptor expressed by the tumour cells (Clezardin et al, 1993; Tuszynski and Nicosia, 1994). Consequently, tumour cells produce very little amounts of TSP-1 in vitro, making it difficult to study the effect of tumour cell-produced TSP-1 in tumour cell biology.

In order to overcome this problem, we used *THBS-1*-transfected breast cancer cells that overexpress TSP-1. We found that, similar to the effect of platelet-produced TSP-1, tumour cell-produced TSP-1 also up-regulates uPAR and uPA expression, uPA and plasmin activities, and promotes tumour cell invasion proportionally to the amount of TSP-1 produced by the tumour cells. The uPA receptor seems to play a key role in this TSP-1-mediated up-regulation in tumour-associated plasmin activity since down-regulation of uPAR with antisense construct in our experiments significantly reduced both uPA and plasmin activities in the TH26 tumour cells. Although tumour cell TSP-1 also up-regulated PAI-1, the overall tumour cell-associated proteolytic activity was significantly up-regulated by tumour cell TSP-1. Moreover, the up-regulation of the plasminogen/plasmin system by tumour cell-produced TSP-1 was significantly higher than the one achieved when we treated tumour cells with platelet-produced TSP-1 at concentrations that mimicked those seen in tumour patients (Arnoletti, 1995; Albo et al, 1997). This could explain the apparent discrepancy between the increase in tumour cell invasion seen in this study and the smaller tumours and lower number of lung metastasis previously reported by Weinstat-Saslow et al in mice injected with the high TSP-1-producing TH26 cells compared to those injected with low TSP-1-producing TH5 cells (Weinstat-Saslow et al, 1994). The excessive amount of TSP-1 expressed by the TH26 cells may promote excessive tumour cell-associated proteolysis. It has been well established that while pericellular proteolysis is fundamental for tumour cell invasion, excessive proteolysis is detrimental for tumour cell invasion in vivo, since it decreases tumour cell proliferation and excessively destroys the matrix scaffold surrounding tumour cells, inhibiting cell adhesion and migration (Krishnamurti, 1992; Hosokawa et al, 1993). In our laboratory, we have previously established that at doses that more closely mimic those seen in cancer patients, platelet-produced TSP-1, through its CSVTCG-specific receptor, promotes tumour cell invasion in vitro and metastasis in a murine model (Wang et al, 1995; 1996*b*; 1996*c*; Albo et al, 1997; Albo, 1998). Alternatively, TSP-1-transfected tumour cells may produce anti-angiogenic factors in vivo which would affect tumour growth. This could explain the lower tumour vascularization and growth seen in mice injected with TH26 cells as reported by Weinstat-Saslow.

Previously, it has been reported that TSP-1 can activate TGF-β1 (Schultz-Cherry and Murphy-Ullrich, 1993; Schultz-Cherry et al, 1994*a*; 1994*b*). TGF-β1 has been shown to up-regulate the plasminogen/plasmin system in other models (Blasi, 1993*a*; 1993*b*). Therefore, in the present study we measured the levels of active TGF-β1 in the conditioned media of the different cell lines. The data showed that active TGF-β1 levels were actually higher in our low TSP-1 producing TH5 cells than in the high TSP-1-producing TH26 cells. This data suggest that the up-regulation seen in the plasminogen/plasmin system in response to tumour TSP-1 seen in our study is not mediated by TGF-β1.

 © 2000 Cancer Research Campaign

In previous experiments, we established that neutralization of uPAR or uPA with antibodies reduced the ability of different tumour cells to invade in response to platelet-produced TSP-1 (Albo et al, 1997). Although these data were convincing, potential pitfalls associated with antibody specificity, availability, and the possibility of cross-linking and spurious activation of receptors and signaling pathways existed. For these reasons, in addition to the antibody experiments, we employed two additional strategies. First, we reduced uPAR expression on the cell surface by enzymatically removing it with GPI-specific phospholipase C. Second and more specifically, we blocked uPAR expression at the mRNA level by antisense inhibition. Additionally, in order to determine whether generation of plasmin at the tumour cell surface was necessary, we inhibited tumour cell surface plasminogen binding with the lysine analogue ε-aminocaproic acid.

The data showed that neutralization of uPAR (with antibody) or enzymatic cleavage of uPAR (with GPI-specific phospholipase C) similarly blocked TSP-1 mediated tumour cell invasion. Although there is the theoretical possibility that other GPI-anchored surface proteins could have been responsible for this effect, the antisense data confirmed the role of uPAR in TSP-1-mediated tumour cell invasion. Although no change in total uPA production was observed in the antisense uPAR-transfected cells, a reduction on cell-associated uPA antigen, uPA activity and plasmin activity that paralleled the reduction in uPAR expression was observed. These data strongly suggested that the main role of uPAR in TSP-1 mediated tumour cell invasion was to promote plasmin generation at the tumour cell surface. Furthermore, ε-aminocaproic acid inhibited TSP-1-mediated tumour cell invasion demonstrating that the generation of plasmin at the tumour cell surface is necessary for the TSP-1-mediated and uPAR-controlled tumour cell invasion to occur. The co-localization of enzyme and substrate at the cell surface favored by TSP-1 allows for an efficient and localized generation of plasmin at the tumour cell surface. Thus, pericellular proteolysis and, therefore, tumour cell invasion are promoted in response to TSP-1.

Tumour cell adhesion plays a crucial role in tumour cell invasion. For tumour cells to metastasize, they must be able to detach from the primary tumour and migrate. Plasmin-mediated degradation of the extracellular matrix is thought to be a prerequisite for tumour cell migration. Although loose attachments to the extracellular matrix are necessary for tumour cells to migrate, it is believed that the overall adhesive capacity of tumour cells is decreased at invasive foci (Mignatti, 1993). uPAR-bound uPA has been shown to be located at tumour cell-substratum focal contact sites, as well as in areas of tumour cell-cell contact (Pollanen, 1987), co-localizing with the intracellular protein vinculin, a specific marker of such contact sites (Hebert, 1988; Pollanen, 1988). uPAR-bound uPA has also been shown to play a crucial role in cell migration (Busso et al, 1994). Our data show that up-regulation of uPAR by tumour cell-produced TSP-1 decreases the overall adhesive capacity of the tumour cells to the main components of the extracellular matrix (Type I collagen) and basement membranes (Type IV collagen and laminin). Furthermore, our data also show that this uPAR-mediated decrease in tumour cell adhesion depends on plasmin generation at the tumour cell surface.

Our collective data suggest a crucial role for TSP-1 in the process of tumour cell invasion, both at the tissue and haematogenous phases. Blocking the plasminogen/plasmin system at different levels could provide for different, simultaneous and, perhaps, synergistic approaches for reducing tumour cell invasion.

### ACKNOWLEDGEMENTS

The authors wish to thank Dr Liliana Ossowski, Department of Medicine, Division of Neoplastic Diseases, Mount Sinai School of Medicine, New York, USA, for providing the uPAR constructs. This work was supported in part by grants CA65675, CA69722 to GPT from the National Institutes of Health and a grant to GPT from the InKine Pharmaceutical, Company, Inc.

### REFERENCES

Albini A, Iwamoto Y, Kleinman H, Martin G, Aaronson S and Kozlowski J (1987) A rapid in vitro assays for quantitating the invasive potential of tumour cells. *Cancer Res* **47**: 3239–3245

Albo D, Arnoletti JP, Castiglioni A, Granick MS, Solomon MP, Rothman VL and Tuszynski GP (1994) Thrombospondin (TSP) and transforming growth factor beta 1 (TGF-beta) promote human A549 lung carcinoma cell plasminogen activator inhibitor type 1 (PAI-1) production and stimulate tumour cell attachment in vitro. *Biochem Biophys Res Commun* **203**: 857–865

Albo D, Berger DH, Wang TN, Hu X, Rothman V and Tuszynski, GP (1997) Thrombospondin-1 and transforming growth factor-beta 1 promote breast tumour cell invasion through up-regulation of the plasminogen/plasmin system. *Surgery* **122**: 493–499

Albo D, Berger DH and Tuszynski GP (1998) The effect of thrombospondin-1 and TGF-beta 1 on pancreatic cancer cell invasion. *J Surg Res* **76**: 86–90

Arnoletti JP, Albo D, Jhala N, Granick MS, Solomon MP, Atkinson B, Rothman VL and Tuszynski GP (1994) Computer-assisted imaged analysis of tumour sections for a new thrombospondin receptor. *Am J Surg* **168**: 433–436

Arnoletti J, Albo D, Granick MS, Solomon MP, Castiglioni A, Rothman VL and Tuszynski GP (1995) Thrombospondin and transforming growth factor-beta 1 increase expression of urokinase-type plasminogen activator and plasminogen activator inhibitor-1 in human MDA-MB-231 breast cancer cells. *Cancer* **76**: 998–1005

Barnathan E, Kuo A, Kariko K, Rosenfeld L, Murray SC, Behrendt N, Ronne E, Weiner E, Hemkin J and Cines DB (1990) Characterization of human endothelial cell urokinase-type plasminogen activator receptor protein and messenger RNA. *Blood* **76**: 1795–1806

Bianchi E, Cohen RL, Dai A, Thor AT, Shuman MA, and Smith HS (1995) Immunohistochemical localization of the plasminogen activator inhibitor 1 in breast cancer. *Int J Cancer* **60**: 597–603

Blasi F, Behrendt N, Cubellis MV, Ellis V, Lund LR and Masucci MT (1990) The urokinase receptor and regulation of cell surface plasminogen activation. *Cell Diff Development* **32**: 247–254

Blasi F (1993*a*) Molecular mechanisms of protease-mediated tumour invasiveness. *J Surg Oncol* **3** (suppl): 21–23

Blasi F (1993*b*) Urokinase and urokinase receptor: a paracrine/autocrine system regulating cell migration and invasiveness. *BioEssays* **15**: 105–111

Busso N, Masur SK, Lazega D, Waxman S and Ossowski L (1994) Induction of cell migration by pro-urokinase binding to its receptor: possible mechanism for signal transduction in human epithelial cells. *J Cell Biol* **126**: 259–70

Clezardin P, Serre C-M, Trzeciak M-C, Drouin J and Delmas PD (1991) Thrombospondin binds to the surface of human osteosarcoma cells and mediates platelet-osteosarcoma cell interaction. *Cancer Res* **51**: 2621–2627

Clezardin P, Frappart L, Clerget M, Pechoux C and Delmas PD (1993) Expression of thrombospondin (TSP1) and its receptors (CD36 and CD51) in normal, hyperplastic, and neoplastic human breast. *Cancer Res* **53**: 1421–1430

Costantini V, Sidoni A, Deveglia R, Cazzato OA, Bellezza G, Ferri I, Bucciarelli E and Nenci GG (1996) Combined overexpression of urokinase, urokinase receptor, and plasminogen activator inhibitor-1 is associated with breast cancer progression: an immunohistochemical comparison of normal, benign, and malignant breast tissues. *Cancer* **77**: 1079–1088

Ellis V, Behrendt N and Dano K (1991) Plasminogen activation by receptor bound urokinase. A kinetic study with both cell-associated and isolated receptor. *J Biol Chem* **266**: 12752–12758

Foekens JA, Schmitt M, Van Putten WL, Peters HA, Kramer MD and Janicke F (1992) Prognostic value of urokinase-type plasminogen activator in 671 primary breast cancer patients. *Cancer Res* **52**: 6101–6105

Graeff H, Harbeck N, Pache L, Wilhelm O, Janicke F and Schmitt M (1992) Prognostic impact and clinical relevance of tumour associated proteases in breast cancer. *Fibrinolysis* **6**: 45–53

© 2000 Cancer Research Campaign 

Grondahl-Hansen J, Hilsenbeck SG, Christensen IJ, Clark GM, Osborne CK and Brunner N (1997) Prognostic significance of PAI-1 and uPA in cytosolic extracts obtained from node-positive breast cancer patients. *Breast Cancer Res Treat* **43**: 153–163

Grossfeld GD, Ginsberg DA, Stein JP, Bochner BH, Esrig D, Groshen S, Dunn M, Nichols PW, Taylor CR, Skinner DG and Cote RJ (1997) Thrombospondin-1 expression in bladder cancer: association with p53 alterations, tumour angiogenesis, and tumour progression. *J Natl Cancer Inst* **89**: 219–227

Hajjar KA (1995) Cellular receptors in the regulation of plasmin generation. *Thromb Haemost* **74**: 294–301

Hebert C and Baker JB (1988) Linkage of extracellular plasminogen activator to the fibroblast cytoskeleton: colocalization of cell surface urokinase with vinculin. *J Cell Biol* **106**: 1241–1247

Hosokawa T, Muraishi A, Rothman V, Papale M and Tuszynski GP (1993) The effect of thrombospondin on invasion of fibrin gels by human A549 lung carcinoma. *Oncology Res* **5**: 183–189

Kariko K, Kuo A, Boyd D, Okada SS, Cines DB and Barnathan ES (1993) Overexpression of urokinase receptor increases matrix invasion without altering cell migration in a human osteosarcoma cell line. *Cancer Res* **53**: 3109–3117

Keski-Oja J, Koli K, Lohi L and Laiho M (1991) Growth factors in the regulation of the plasminogen plasmin-system in tumour cells. *Semin Thromb Hemost* **17**: 231–239

Kook YH, Adamski J, Zelent A and Ossowski L (1994) The effect of antisense inhibition of urokinase receptor in human squamous cell carcinoma on malignancy. *EMBO J* **13**: 3983–3991

Krishnamurti C and Alving B (1992) Plasminogen activator inhibitor type 1: biochimestry and evidence for modulation of fibrinolysis in vivo. *Semin Thromb Hemost* **18**: 67–80

Laiho M and Keski-Oja J (1989) Growth factors in the regulation of pericellular proteolysis. *Cancer Res* **49**: 2533–2553

Mignatti P and Rifkin DB (1993) Biology and biochemistry of proteinases in tumour cell invasion. *Physiol Rev* **73**: 161–195

Mohanam S, Sawaya R, McCutcheon I, Ali-Osman F, Boyd D and Rao JS (1993) Modulation of in vitro invasion of human glioblastoma cells by urokinase-type plasminogen activator receptor antibody. *Cancer Res* **53**: 4143–4147

Mohanam S, Sawaya RE, Yamamoto M, Bruner JM, Nicholson GL and Rao JS (1994) Proteolysis and invasiveness of brain tumours: role of urokinase-type plasminogen activator receptor. *J Neurooncol* **22**: 153–160

Nathan FE, Hernandez E, Dunton CJ, Treat J, Switalska HI, Joseph RR and Tuszynski GP (1994) Plasma thrombospondin levels in patients with gynecologic malignancies. *Cancer* **73**: 2853–2858

Ossowski L, Clunie G, Masucci MT and Blasi F (1991) In vivo paracrine interaction between urokinase and its receptor: effect on tumour cell invasion. *J Cell Biol* **115**: 1107–1112

Ossowski L (1992) Invasion of connective tissue by human carcinoma cell lines: requirement for urokinase, urokinase receptor, and interstitial collagenase. *Cancer Res* **52**: 6754–6760

Ploug M, Kjalke M, Ronne E, Weidle U, Hoyer-Hansen G and Dano K (1993) Localization of the disulfide bonds in the NH2-terminal domain of the cellular receptor for human urokinase-type plasminogen activator. A domain structure belonging to a novel superfamily of glycolipid-anchored membrane proteins. *J Biol Chem* **268**: 17539–17546

Plow E, Freaney DE, Plescia J and Miles LA (1986) The plasminogen system and cell surfaces: evidence for plasminogen and urokinase receptors on the same cell type. *J Cell Biol* **103**: 2411–2420

Pollanen J, Saksella O, Salonen EM, Andreasen P, Nielsen L, Dano K and Vaheri A (1987) Distinct localizations of urokinase-type plasminogen activator and its type 1 inhibitor under cultured human fibroblasts and sarcoma cells. *J Cell Biol* **104**: 1085–1096

Pollanen J, Hedman K, Nielsen LS, Dano K and Vaheri A (1988) Ultrastructural localization of plasma-membrane associated urokinase-type plasminogen activator at focal contacts. *J Cell Biol* **106**: 87–95

Roldan A, Cubellis MV, Masucci N, Behrendt LR, Lund K, Dano E, Appela E and Blasi F (1990) Cloning and expression of the receptor for human urokinase plasminogen activator, a central molecule in cell surface, plasmin dependent proteolysis. *EMBO J* **9**: 467–474

Roth JJ, Reiver DM, Granick MS, Rothman VL, Nicosia RF and Tuszynski GP (1997) Histopathology and clinical assessment correlate with the cysteine-serine-valine-threonine-cysteine-glycine (CSVTCG) receptor of thrombospondin-1 in breast tumours. *Histol Histopathol* **12**: 1013–1018

Schultz-Cherry S and Murphy-Ullrich JE (1993) Thrombospondin causes activation of latent transforming growth factor-β secreted by endothelial cells by a novel mechanism. *J Cell Biol* **122**: 923–932

Schultz-Cherry S, Lawler J and Murphy-Ullrich JE (1994*a*) The type 1 repeats of thrombospondin 1 activate latent transforming growth factor-β. *J Biol Chem* **269**: 26783–26788

Schultz-Cherry S, Ribeiro S, Gentry L and Murphy-Ullrich, JE (1994*b*) Thrombospondin binds and activates the small and large forms of latent transforming growth factor-β in a chemically defined system. *J Biol Chem* **269**: 26775–26782

Seiffert D, Ciambrone G, Wagner NV, Binder BR and Loskutoff DJ (1994) The somatomedin B domain of vitronectin. Structural requirements for the binding and stabilization of active type I plasminogen activator inhibitor. *J Biol Chem* **269**: 2659–2666

Testa JE and Quigley JP (1990) The role of urokinase-type plasminogen activator in aggressive tumour cell behavior. *Cancer Metastasis Rev* **9**: 353–367

Tuszynski GP (1993) The role of thrombospondin in malignancy and tumour cell metastasis. In: *Thrombospondin*, Lahav J (ed) pp. 209–225. CRC Press: Boca Raton

Tuszynski GP and Murphy A (1990) Spectrophotometric quantitation of anchorage-dependent cell numbers using the bicinchoninic acid protein assay reagent. *Anal Biochem* **184**: 189–191

Tuszynski GP and Nicosia RF (1994) Localization of thrombospondin and its cysteine-serine-valine-threonine-cysteine-glycine-specific receptor in human breast carcinoma. *Lab Invest* **70**: 228–233

Tuszynski GP and Nicosia RF (1996) The role of thrombospondin-1 in tumour progression and angiogenesis. *BioEssays* **18**: 71–76

Tuszynski GP, Gasic TB, Rothman VL, Knudsen KA and Gasic GJ (1987*a*) Thrombospondin, a potentiator of tumour cell metastasis. *Cancer Res* **47**: 4130–4133

Tuszynski GP, Rothman V, Murphy A, Siegler K, Smith L, Smith S, Karczewski J and Knudsen KA (1987*b*) Thrombospondin promotes cell-substratum adhesion. *Science* **236**: 1570–1573

Tuszynski GP, Switalska HI and Knudsen K (1987*c*) Methods of studying platelet-secreted proteins and the platelet cytoskeleton. *Mod Meth Pharm* **4**: 267–286

Tuszynski GP, Smith M, Rothman VL, Capuzzi DM, Joseph RR, Katz J, Besa EC, Treat J and Switalska HI (1992) Thrombospondin levels in patients with malignancy. *Thromb Haemost* **67**: 607–611

Tuszynski GP, Wang TN and Berger D (1997) Adhesive proteins and the hematogenous spread of cancer. *Acta Haematologica* **97**: 29–39

Vassalli J, Baccino D and Belin D (1985) A cellular binding site for the Mr 55,000 form of the human plasminogen activator, urokinase. *J Cell Biol* **100**: 86–92.

Vassalli J, Sappino AP and Belin D (1991) The plasminogen activator/plasmin system. *J Clin Invest* **88**: 1067–1072

Wang TN, Qian X, Granick MS, Solomon MP, Rothman VL and Tuszynski GP (1995) The effect of thrombospondin (TSP) on oral squamous carcinoma cell invasion of collagen. *Am J Surg* **170**: 502–505

Wang TN, Qian X, Granick MS, Solomon MP, Rothman VL, Berger DH and Tuszynski GP (1996*a*) Thrombospondin-1 (TSP-1) promotes the invasive properties of human breast cancer. *J Surg Res* **63**: 39–43

Wang TN, Qian XH, Granick MS, Solomon MP, Rothman VL, Berger DH and Tuszynski GP (1996*b*) Inhibition of breast cancer progression by an antibody to a thrombospondin-1 receptor. *Surgery* **120**: 449–454

Wang TN, Qian XH, Granick MS, Solomon MP, Rothman VL, Berger DH and Tuszynski GP (1996*c*) Thrombospondin-1 (TSP-1) promotes the invasive properties of human breast cancer. *J Surg Res* **63**: 39–43

Weinstat-Saslow DL, Zabrenetzky VS, VanHoutte K, Frazier WA, Roberts DD and Steeg PS (1994) Transfection of thrombospondin 1 complementary DNA into a human breast carcinoma cell line reduces primary tumour growth, metastatic potential, and angiogenesis. *Cancer Res* **54**: 6504–6511

Wong SY, Purdie AT and Han P (1992) Thrombospondin and other possible related matrix proteins in malignant and benign breast disease: An immunohistochemical study. *Am J Pathol* **140**: 1473–1482

Yabkowitz R, Mansfield PJ, Dixit VM and Suchard SJ (1993) Motility of human carcinoma cells in response to thrombospondin: Relationship to metastatic potential and thrombospondin structural domains. *Cancer Res* **53**: 378–387

Yamashita Y, Kurohiji T, Tuszynski GP, Sakai T and Shirakusa T (1998) Plasma thrombospondin levels in patients with colorectal carcinoma. *Cancer* **82**: 632–638

Yu W, Kim J and Ossowski L (1997) Reduction in surface urokinase receptor forces malignant cells into a protracted state of dormancy. *J Cell Biol* **137**: 767–777

 *© 2000 Cancer Research Campaign*
